# Thermal Hazard Analysis of Two Non-Ideal Explosives Based on Ammonium Perchlorate/Ammonium Nitrate and Aluminium Powder

**DOI:** 10.3390/molecules29112680

**Published:** 2024-06-05

**Authors:** Jiahu Guo, Xiaoping Chen, Yanwu Yu, Jianhui Dong, Jun Zhang, Jingwei Meng, Chenglai Xin, Zhigang Wang

**Affiliations:** 1 Emergency Management College, Chengdu University, Chengdu 610106, China; guojiahu@cdu.edu.cn (J.G.); xinchenglai@cdu.edu.cn (C.X.); wangzhigang@cdu.edu.cn (Z.W.); 2 School of Environment and Safety Engineering, North University of China, Taiyuan 030051, China; 3 Ordnance Engineering College, Naval Engineering University, Wuhan 430030, China; 4 Experimental Center of Advanced Materials, School of Materials Science & Engineering, Beijing Institute of Technology, Beijing 100081, China; 7520210155@bit.edu.cn

**Keywords:** hazardous materials, thermal hazard, oxygen balance, kinetic parameters, storage safety, emergency prevention

## Abstract

In recent years, various kinds of civil explosive detonation accidents have occurred frequently around the world, resulting in substantial human casualties and significant property losses. It is generally believed that thermal stimulation plays a critical role in triggering the detonation of explosives; consequently, the study of the thermal hazards of explosives is of great significance to many aspects of safety emergency management practices in the production, transportation, storage, and use of explosives. It is known that the thermal stability of the ammonium perchlorate-aluminium system and the ammonium nitrate-aluminium system has been extensively investigated previously in the literature. However, there is a paucity of research on the thermal hazard characteristics of non-ideal explosives under varying oxygen balance conditions within the academic sphere. Therefore, this research focused on the study of the thermal hazards of non-ideal explosives based on thermokinetic analysis. The thermal hazards of non-ideal explosive mixtures of ammonium perchlorate and aluminium and of ammonium nitrate and aluminium were studied by thermal analysis kinetics. The thermokinetic parameters were meticulously studied through differential scanning calorimetry (DSC) analysis. The results showed that the peak reaction temperature and activation energy of the ammonium perchlorate-aluminium system were significantly higher than those of the ammonium nitrate-aluminium system. Under the condition of zero oxygen balance, the peak reaction temperature of the ammonium nitrate-aluminium system was 259 °C (heating rate 5 °C/min), and the activation energy was 84.7 kJ/mol. Under the same conditions, the peak reaction temperature and activation energy of the ammonium perchlorate-aluminium system were 292 °C (heating rate 5 °C/min) and 94.9 kJ/mol, respectively. These results indicate that the ammonium perchlorate-aluminium system has higher safety under the same thermal stimulation conditions. Furthermore, research on both non-ideal explosive systems reveals that the activation energy is at its peak under negative oxygen balance conditions, recorded at 104.2 kJ/mol (ammonium perchlorate-aluminium) and 86.2 kJ/mol (ammonium nitrate-aluminium), which indicates a higher degree of safety. Therefore, the investigation into the thermal hazards of non-ideal explosive systems under different oxygen balance conditions is of utmost importance for the enhancement and improvement of safety emergency management practices.

## 1. Introduction

Ammonium perchlorate and ammonium nitrate, serving as oxidizers, and metallic powders such as aluminium and magnesium powders, acting as high-energy fuels, are extensively used in the formulation of solid composite propellants, high-energy explosives, and civil explosives [1,2,3]. However, improper handling and disposal of these substances (explosives) during their production, transportation, storage, and application may lead to severe accidents such as combustion and detonation, resulting in substantial casualties and significant property damages and posing a grave threat to people’s daily activities, livelihoods, and social stability [4]. On 4 August 2020, an ammonium nitrate accident in the Port of Beirut took the lives of at least 190 people while injuring 6500. On 12 August 2015, a fire-triggered ammonium nitrate accident in the Binhai New Area of Tianjin caused 165 killed, 8 missing, 798 injured, and direct economic losses amounting to CNY 6.866 billion. In order to reduce the probability of accidents and minimize losses, researchers around the world are taking a keen interest in research into the hazards associated with explosive storage [5,6,7,8]. With the advancement of military technology and the invention of various sophisticated weapons, soldiers have to adapt themselves to a more complex and volatile battleground, and ammunition survivability also faces tremendous threats. When accidentally stimulated by bullet fragments, fire, shock waves, electromagnetic radiation, or high-speed jets, ammunition can lead to very serious accidents [9,10,11].

In explosives containing aluminium and ammonium perchlorate (AP), the detonation process is characterized as non-ideal detonation due to the participation of aluminium powder and AP in the detonation reaction. Consequently, such composite explosives are categorized as non-ideal explosives. Civil explosives, also known as industrial explosives, fall within the scope of non-ideal explosives. Non-ideal explosives are highly flammable and blastable, so there are risks of combustion and explosion in the whole course, including design, experiment, preparation, production, transportation, storage, use, and disposal. As ammunition has a specified thermal sensitivity, there are certain risks of explosion both on the battlefield and in regular storage or conveyance [12,13,14,15]. Related studies show that among the aforesaid destabilizing factors, heat is the most common form of energy that stimulates explosives and causes the most accidents. That explains why studying the thermal hazards of non-ideal explosives is of great consequence to their production, storage, and application [13,14,15,16,17]. However, to the best of our knowledge, studies exploring the thermal hazard traits of non-ideal explosives under different oxygen balance conditions are notably scarce within academic research. In this paper, two non-ideal explosives, one as a mixture of ammonium perchlorate and aluminium powder and the other as a mixture of ammonium nitrate and aluminium, are studied to observe their thermal behaviors under the nitrogen condition based on thermal analysis kinetics under various oxygen balance conditions (positive oxygen balance, zero oxygen balance, and negative oxygen balance). Besides, the research calculates the kinetic parameters of the two explosives.

## 2. Experiment

### 2.1. Reagents and Instruments

The reagents include ammonium perchlorate (NH_4_ClO_4_, purity: 99.7%, supplied by Tianjin Guangfu Fine Chemical Research Institute, Tianjin, China), ammonium nitrate (NH_4_NO_3_, purity: 99.7%, supplied by Beijing Branch of Sinopharm Chemical Reagent Co., Ltd., Beijing, China), and aluminium powder (purity: 99.9%, supplied by Ansteel Group Aluminium Powder Co., Ltd., Anshan, China). Prior to testing, grind the NH_4_ClO_4_ and NH_4_NO_3_ samples into fine powder and heat them dry for two hours at 70 °C, heat aluminium powder for two hours at 100 °C, and pass the samples through a 400-mesh sieve before testing.

The instruments include an AL 201-IC Mettler Toledo electronic scale (made by Mettler Toledo Technologies (China) Co., Ltd., Shanghai, China), a DHG303-2 constant-temperature drying oven (made by Shanghai Bluepard Instruments Co., Ltd., Shanghai, China), and an HTC-1 differential thermal balance (made by Beijing Hengjiu Experimental Equipment Co., Ltd., Beijing, China).

### 2.2. Determination of Experimental Formulation

Oxygen balance (OB) is an important indicator used for explosives measurement and refers to the excess or inadequate amount of oxygen left after the oxygen contained in the explosive is fully used to oxidize the combustible elements [4]. In this experiment, the test sample formulations of interest are determined based on the respective computations of zero OB, negative OB, and positive OB for the combination of the selected strong oxidizer and reducer. OB computations for an explosive mixture are expressed in terms of the algebraic sum of the product of the OB value of each component and its mass percent, as shown in Formula (1):

(1) $$OB=\sum_{i}OB_{i}w_{i}$$

where *OB_i_* is the OB value of the *i*th component of the explosive mixture; and *w_i_* is the mass percent of the *i*th component of the explosive mixture.

Based on the OB theory, we can determine the following formulation of the non-ideal explosives in Table 1.

### 2.3. Test Sample Preparation and Thermal Analysis Experiment

Commensurate amounts of oxidizer and reducer are measured out and transferred onto the electronic scale based on the formulations of the non-ideal explosives in Table 1. Then, weigh the finally prepared samples of non-ideal explosives with the weighing paper. For each experiment, use a dry spoon to transfer 10 mg ± 0.1 mg into the alumina crucible. The heating rate of the thermal analysis experiment includes 5 °C/min, 10 °C/min, and 20 °C/min and the experimental temperature range is set at 10–500 °C. The experimental gas is nitrogen at a flow rate of 50 mL/min. The initial heating rate is set at 0, and the heating time is set at 5 min in order to ensure thorough nitrogen replacement.

## 3. Thermokinetic Analysis Methods

In the past decade, thermal analysis has been widely used in a wide range of fields, including inorganic substances [18], pigments [19], explosives [20,21,22], energetic materials [23,24,25,26], polymers [27,28,29,30,31,32], nanocomposites [33], and biomass materials [34,35]. The main techniques used for sample measurements are differential scanning calorimetry (DSC), thermogravimetric analysis (TGA), and dynamic mechanical analysis (DMA). In special cases, combined on-line techniques such as evolved gas analysis and visual monitoring have also been employed. In this study, the authors utilized DSC to analyze the thermal hazard characteristics of two non-ideal explosives based on ammonium perchlorate/ammonium nitrate, and aluminium powder.

As there are some new mathematical approximations, it seems that the Ozawa and Kissinger methods have not been so popular in the International Confederation for Thermal Analysis and Calorimetry (ICTAC) Kinetics Practices papers in recent years [36,37,38]. However, many researchers still perform thermokinetic parameter calculations employing the Ozawa and Kissinger methods due to the unique advantages of the two methods [39,40,41,42,43,44,45], which means that these two approximation methods are still reliable methods. Therefore, the authors adopt the Ozawa and Kissinger methods in this study to analyze the thermokinetic parameters such as activation energy (E) and pre-exponential factor (A).

### 3.1. The Ozawa Method Based on Integral Calculus

In thermokinetic analysis, the Ozawa method has a remarkable advantage in that it can work out the value of activation energy (E) directly without the selection of the mechanism function, therefore avoiding any potential error attributable to the hypothetical differences as to the mechanism function. In this paper, the Ozawa function is used to compute E-values under various OB conditions, as shown in Formula (2):

(2) $$\mathrm{lg}\beta =\mathrm{lg}\left(\frac{AE_{a}}{RG(a)}\right)-2.315-0.4567\frac{E_{a}}{RT}$$

where *β* is the heating rate; *A* is the pre-exponential factor; *E*_a_ is activation energy; *R* is the molar gas constant (8.314 J·mol^−1^·K^−1^); and *T* is the peak temperature (*K*). *G*(a) is a constant as the same a-value is chosen under different heating rates (*β_i_*). Since lg*β* is linear with 1/*T*, simple linear fitting can be performed to work out the *E*_a_-value based on the slope.

### 3.2. The Kissinger Method Based on Differential Calculus

The Kissinger method, which hypothesizes that the mechanism function conforms to the nth reaction model, has its advantage in that it can work out the values of activation energy (E) and the pre-exponential (A). The Kissinger function is as shown in Formula (3):

(3) $$\ln\left(\frac{\beta_{i}}{T_{pi}^{2}}\right)=\ln\frac{A_{k}R}{E_{k}}-\frac{E_{k}}{R}\frac{1}{T_{pi}}\left[i=1,\;2,\;3,\cdots \right]$$

where *β_i_* is the heating rate; *A*_k_ is the pre-exponential factor; *E*_k_ is activation energy; *R* is the molar gas constant (8.314 J·mol^−1^·K^−1^); and *T*_p*i*_ is the peak temperature (*K*).

A simple linear fit of lg(*β_i_*/*T*_p*i*_^2^) vs. 1/*T*_p*i*_ gives us a straight line, from the slope of which we can work out activation energy (*E*_k_). Based on the intercept, we can work out the *A*_k_-value.

## 4. Experimental Results and Discussion

### 4.1. Results of the Thermal Analysis Experiment of the Ammonium Perchlorate-Aluminium System

**Figure 1:**
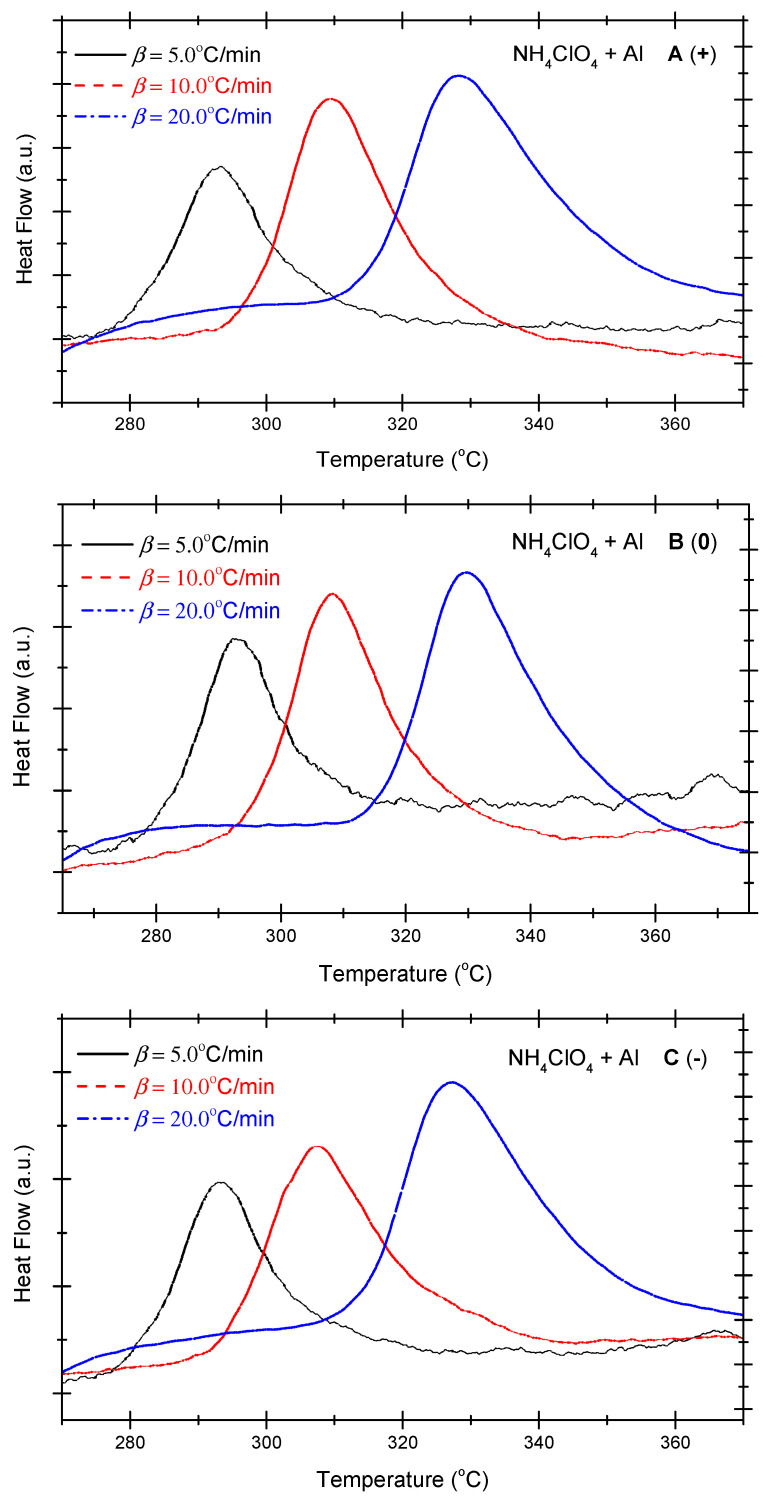
DSC curves of ammonium perchlorate-aluminium system ((**A**) (+): positive oxygen; (**B**) (0): zero oxygen; (**C**) (−): negative oxygen). (Note: The black, red, and blue dotted lines in the above figure correspond to heating rates of 5 °C/min, 10 °C/min and 20 °C/min, respectively).

Figure 1 presents the differential scanning calorimetry (DSC) curves of the non-ideal explosive composed of ammonium perchlorate and aluminium powder. As shown in Figure 1, under positive OB, zero OB, and negative OB conditions, the DSC curves of non-ideal explosives composed of ammonium perchlorate and aluminium powder tend to be similar. In other words, with the increase in the heating rate, the exothermic peak temperatures of the three curves incline towards high levels. Besides, as the exothermic peak temperature becomes steeper with the increase in the heating temperature, the total heat release of the system mounts gradually. Table 2 summarizes the characteristic peak temperatures of the DSC curves of the ammonium perchlorate-aluminium powder system at different heating temperatures. A seen in Table 2, all peak temperatures (T_p_) incline to a higher level as the *β*-value increases. In order to quantitatively analyze the phenomenon, we can compute the thermokinetic parameters of the non-ideal explosive composed of ammonium perchlorate and aluminium powder under different OB conditions.

### 4.2. Thermokinetic Parameters of the Ammonium Perchlorate-Aluminium System

**Figure 2:**
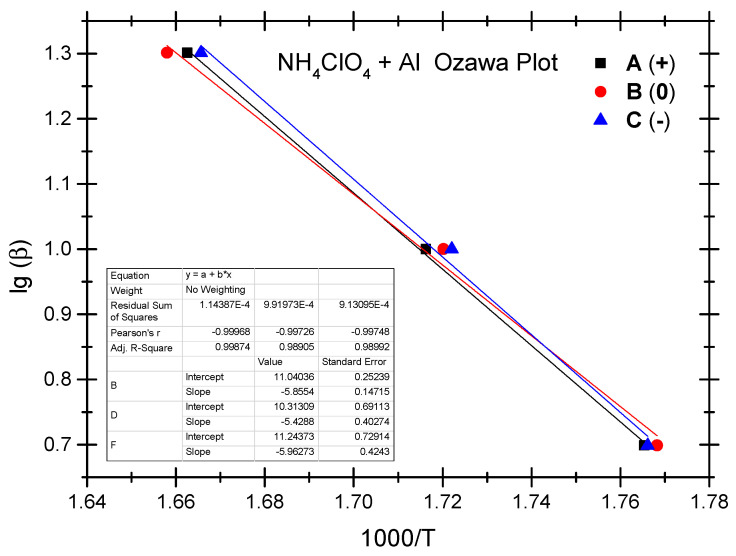
Ozawa fitting diagram of ammonium perchlorate-aluminium system.

**Figure 3:**
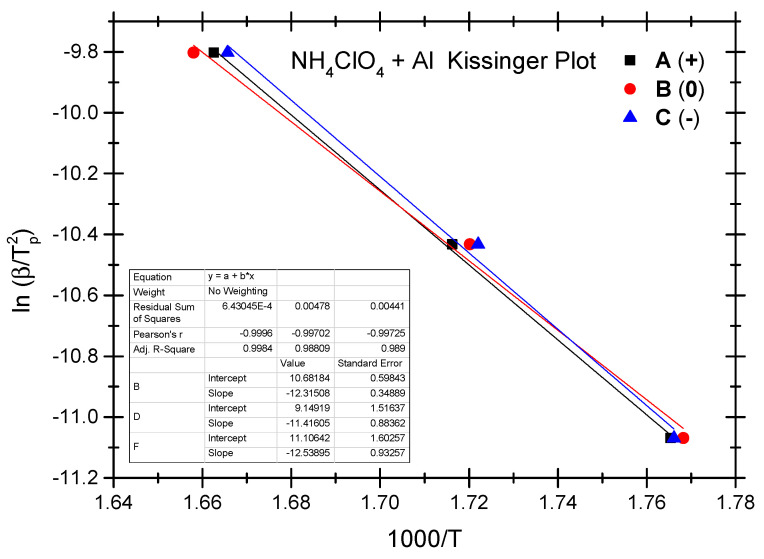
Kissinger fitting diagram of ammonium perchlorate-aluminium system.

The Ozawa method and the Kissinger method are used to calculate the thermokinetic parameters of the ammonium perchlorate-aluminium system under different OB conditions. Ozawa fitting is performed using the peak temperatures (T_p_) corresponding to the different heating rates (*β*). As shown in Figure 2, a simple linear fit of 1 g*β* vs. 1000/T_p_ is carried out. Similarly, Figure 3 presents the simple linear fit of ln(*β*/T_p_^2^) vs. 1000/T_p_ based on the relevant peak temperature data and the Kissinger method. As seen in Figure 2 and Figure 3, the fitted line shows a very good linear correlation. The thermokinetic parameters of the ammonium perchlorate-aluminium system are computed using the Ozawa function (2) and the Kissinger function (3), respectively, as shown in Table 3.

As indicated by the data in Table 3, the non-ideal ammonium perchlorate-aluminium system has an activation energy (*E*_a_-value) between 98.8 kJ/mol and 108.5 kJ/mol based on the Ozawa method, while the activation energy (*E*_k_-value) calculated based on the Kissinger method stands at 94.9–104.2 kJ/mol. This means that the pre-exponential factor value stands between 1.07 × 10^5^ and 8.35 × 10^5^. There is a slight difference between the two fitting methods as to the activation energy data, and the Ozawa method shows a slightly higher value. The relevant literature reports a similar difference [40,41,42,43,44]. In these studies, researchers found that the activation energy value calculated based on the Kissinger method is slightly higher. It testifies to the fact that the difference in the activation energy value is very common when different methods are used to fit the experimental data; in other words, this difference is not a result of the experimental objects, system, and heating rate but a result of the various fitting calculation methods. As indicated by the experiment, the non-ideal ammonium perchlorate-aluminium system has the lowest activation energy under the zero OB condition, which is a very ideal condition for combustion and explosion and where the combustible components are fully oxidized and combusted; that is why the system is apt to combust and explode when exposed to thermal stimulation. Activation energy becomes the highest under the negative OB condition, indicating that some of the combustible elements fail to combust thoroughly because the explosive does not have enough oxygen to thoroughly oxidize these elements. The probability of combustion and explosion is slightly lower under the negative OB condition than under the ideal zero OB condition.

### 4.3. Results of the Thermal Analysis Experiment of the Ammonium Nitrate-Aluminium System

**Figure 4:**
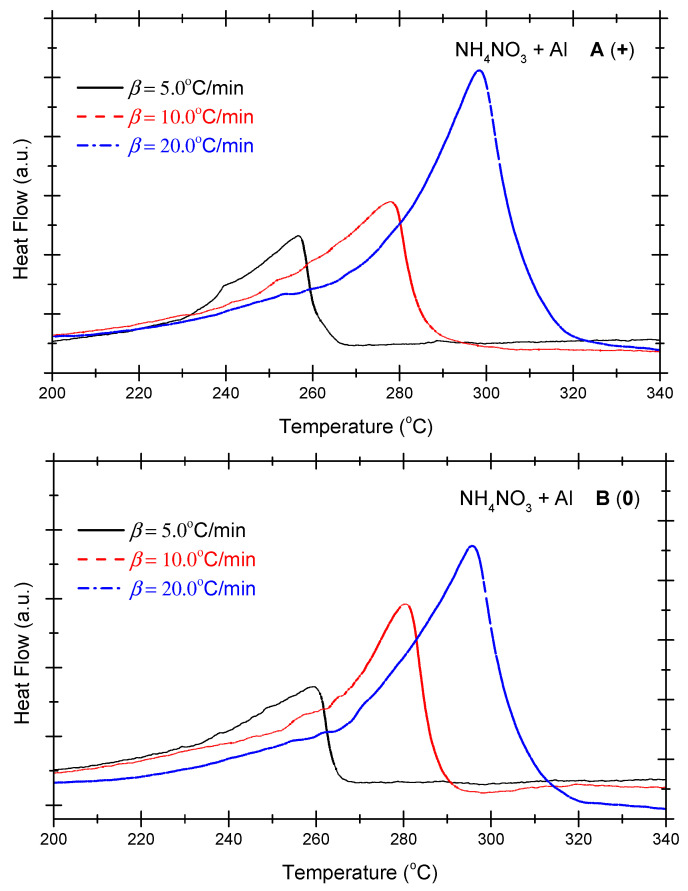

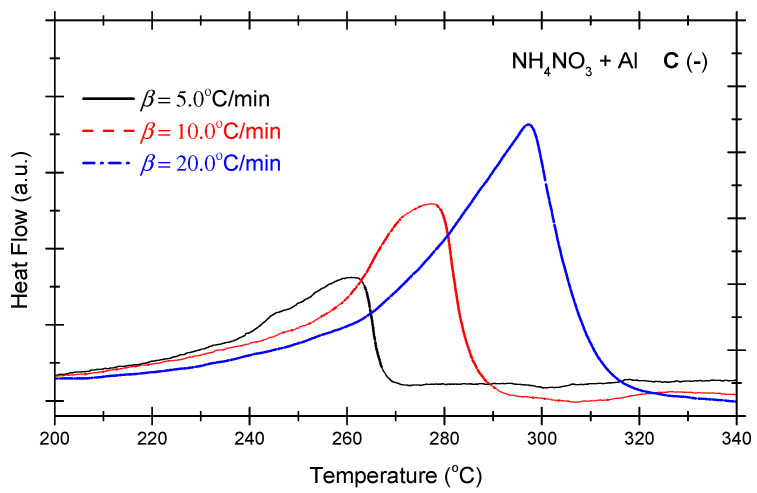
DSC curves of ammonium nitrate–aluminium ((**A**) (+): positive oxygen; (**B**) (0): zero oxygen; (**C**) (−): negative oxygen). (Note: The black, red, and blue dotted lines in the above figure correspond to heating rates of 5 °C/min, 10 °C/min and 20 °C/min, respectively).

Figure 4 presents the DSC curves of the non-ideal explosive composed of ammonium nitrate and aluminium powder under the various OB conditions. As indicated in Figure 4, under positive OB, zero OB, and negative OB conditions, the curves of the non-ideal explosive composed of ammonium nitrate and aluminium powder tend to be similar. In other words, with the increase in the heating rate, the exothermic peak temperatures of the three curves incline towards higher levels. Moreover, as the exothermic peak increases with the heating rate and inclines to become steeper, the heat release of the system mounts gradually. As shown in Figure 2, which deals with the ammonium perchlorate–aluminium system, the ammonium nitrate–aluminium system exhibits a steeper curve, which means the heat release process is more violent and rapid. Table 4 summarizes the characteristic peak temperatures of the DSC curves of the ammonium nitrate-aluminium system at different heating temperatures. As seen in Table 4, all peak temperatures (T_p_) incline to a higher level as the *β*-value increases. In order to quantitatively analyze the phenomenon, we can compute the thermokinetic parameters of the non-ideal explosive composed of ammonium nitrate and aluminium under different OB conditions.

### 4.4. Thermokinetic Parameters of the Ammonium Nitrate-Aluminium System

**Figure 5:**
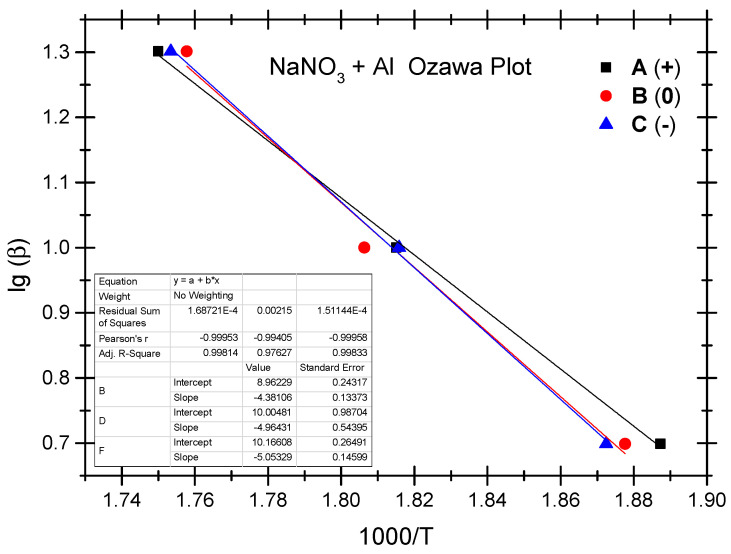
Ozawa fitting diagram of ammonium nitrate-aluminium system.

**Figure 6:**
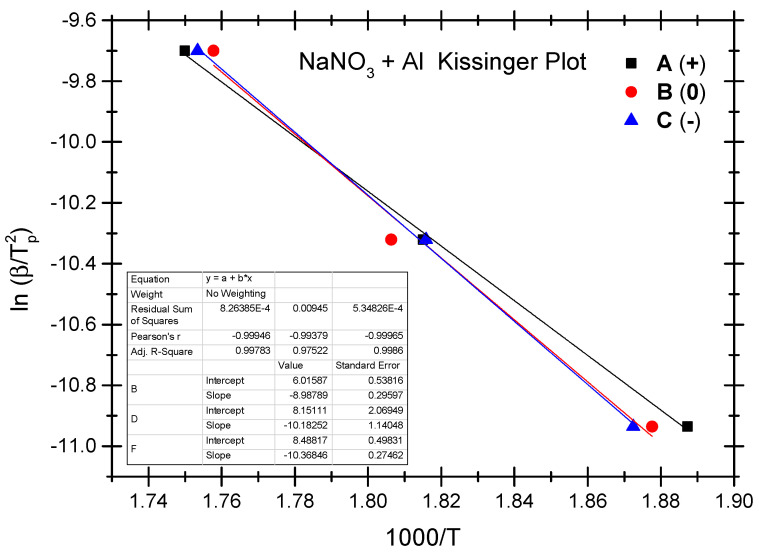
Kissinger fitting diagram of ammonium nitrate-aluminium system.

The Ozawa method and the Kissinger method are used to calculate the thermokinetic parameters of the ammonium nitrate-aluminium system under different OB conditions. In Table 4, Ozawa fitting is conducted using the peak temperatures (T_p_) corresponding to the different heating rates (*β*). As shown in Figure 5, a simple linear fit of 1 g*β* vs. 1000/T_p_ is performed. Similarly, Figure 6 presents the simple linear fit of ln(*β*/T_p_^2^) vs. 1000/T_p_ based on the relevant peak temperature data and the Kissinger method. As seen in Figure 5 and Figure 6, the fitted line shows a very good linear correlation. The thermokinetic parameters of the ammonium nitrate-aluminium system are computed using the Ozawa function (2) and the Kissinger function (3), respectively, as shown in Table 5.

According to the data in Table 5, the activation energy (*E*_a_) of the non-ideal ammonium nitrate-aluminium system calculated based on the Ozawa method stands at 79.8 to 92.0 kJ/mol, while the activation energy (*E*_k_) calculated based on the Kissinger method stands between 74.7 kJ/mol and 86.2 kJ/mol. Furthermore, the value of the pre-exponential factor (A_k_) stands between 3.68 × 10^3^ and 5.04 × 10^4^. There is a slight difference between the two methods as regards activation energy in that the Ozawa method demonstrates slightly higher activation in both the ammonium nitrate-aluminium system and the ammonium perchlorate-aluminium system. The phenomenon is perhaps attributable to different fitting calculation methods. As shown by the experimental results, as with the ammonium perchlorate-aluminium system, the non-ideal ammonium nitrate–aluminium system has the highest activation energy under the negative OB condition. As is different from the perchlorate-aluminium system, the ammonium nitrate-aluminium system has the lowest activation energy under the positive OB condition; this is perhaps attributable to the fact that excess oxygen can make up for oxygen consumption as a result of the production of NO, NO_2_, and other nitrogen oxides during the combustion and explosion of the ammonium nitrate-aluminium system. Another point that cannot be ignored is that, during the decomposition reaction, many parallel and successive reactions take place. Their share changes with the heating rate and the degree of reaction. Furthermore, if we compare the peak temperature and activation energy of the two non-ideal explosives, we will notice that the peak temperature and activation energy of the ammonium nitrate-aluminium system are remarkably lower than those of the ammonium perchlorate-aluminium system. In other words, the perchlorate-aluminium system is more thermally stable, while the ammonium nitrate-aluminium system has more risks of combustion and explosion when exposed to thermal stimulation.

It is worthy to note that some researchers found that the main explosive particle size and the aluminium particle size have significant effects on the thermal stability, critical temperature, explosion heat, and energy output of the explosion, among other parameters [46,47,48,49,50]. The smaller the particle size of the main explosive and aluminium powder, the lower the activation energy of the explosive mixture. In comparison, an explosive mixed with aluminium powder of a larger particle size is more thermally stable [46]. However, in a study carried out by Yao Lina [48], it was found that DNTF (3,4-Bis (3-nitrofurazan-4-yl) furoxan) pressed explosive mixture containing 5% aluminium nanoparticles and 25% aluminium microparticles performs better in thermal stability than DNTF explosive mixture containing 30% aluminium microparticles.

## 5. Conclusions and Prospects

The findings from thermal analysis kinetics experiments manifest that the non-ideal explosive mixture of ammonium nitrate and aluminium exhibits both lower peak temperatures and activation energies compared to that of ammonium perchlorate and aluminium, and the non-ideal explosive mixture of ammonium nitrate and aluminium shows a more abrupt exothermic peak, indicating that ammonium nitrate-aluminium is more susceptible to thermal stimulation, predisposing it to combustion and detonation, conversely the ammonium perchlorate-aluminium system has higher safety under the same thermal stimulation conditions.

The non-ideal explosive mixture of ammonium perchlorate and aluminium displays a relatively lower activation energy under zero oxygen balance (OB) conditions, quantified as 94.9 kJ/mol (as determined by the Kissinger method). Conversely, the non-ideal explosive mixture of ammonium nitrate and aluminium exhibits the lowest activation energy under positive OB conditions, recorded at 74.7 kJ/mol (Kissinger method). This indicates that both non-ideal explosives are more thermally hazardous under positive and zero OB conditions, with an increased propensity for thermally induced combustion and detonation. Research on both non-ideal explosive systems reveals that the activation energy is at its peak under negative OB conditions, with the non-ideal explosive mixtures of ammonium perchlorate and aluminium and of ammonium nitrate and aluminium being 104.2 kJ/mol and 86.2 kJ/mol, respectively (as determined by the Kissinger method). Consequently, it can be inferred that the likelihood of combustion and detonation for these two systems under negative OB conditions is comparatively lower, thereby implying a higher degree of safety.

This study underscores that by adjusting the OB of these two non-ideal explosive systems, it is possible to reduce the risk of combustion and detonation, thereby enhancing their overall safety. Therefore, the investigation into the thermal hazards of non-ideal explosive systems under different OB conditions is of paramount importance for the enhancement and improvement of safety emergency management practices in the production, storage, and transportation of such explosives. 

Furthermore, the authors noticed that not only the oxygen balance but also the particle size, size distribution, and assembly methods of non-ideal explosives will impact the thermal stability of explosives. Therefore, as part of research on the hazardous characteristics of non-ideal explosives, the influence of particle size, size distribution, and assembly methods of non-ideal explosives on the hazardous characteristics may be a field of interest, based on research on the type of explosives and the effect of OB.

## Figures and Tables

**Table 1:** Formulation of non-ideal explosive.

No.	Test Sample	Positive OB A (+)	Zero OB B (0)	Negative OB C (−)
1	NH_4_ClO_4_ + Al	1:0.33	1:0.37	1:0.43
2	NH_4_NO_3_ + Al	1:0.19	1:0.22	1:0.25

**Table 2:** DSC peak temperatures of ammonium perchlorate-aluminium system at different heating rates.

*β* (°C/min)	T_P_ (°C)		
	A (+)	B (0)	C (−)
5.0	293.3	292.4	293.1
10.0	309.5	308.3	307.5
20.0	328.3	330.0	327.2

Note: *β* is the heating rate, T_P_ is the peak temperature, and the same below.

**Table 3:** Kinetic parameters of ammonium perchlorate-aluminium system.

	OB	Ozawa Method		Kissinger Method		
		*E*_a_ (kJ/mol)	R^2^	*E*_k_ (kJ/mol)	R^2^	*A*_k_
A (+)	0.0325	106.6	0.9987	102.4	0.9984	5.36 × 10^5^
B (0)	0.0079	98.8	0.9891	94.9	0.9881	1.07 × 10^5^
C (−)	−0.0290	108.5	0.9899	104.2	0.9890	8.35 × 10^5^

**Table 4:** DSC peak temperatures of ammonium nitrate-aluminium system at different heating rates.

*β* (°C/min)	T_P_ (°C)		
	A (+)	B (0)	C (−)
5.0	256.7	259.4	260.9
10.0	277.8	280.5	277.6
20.0	298.3	295.8	297.2

**Table 5:** Kinetic parameters of ammonium nitrate-aluminium system.

	OB	Ozawa Method		Kissinger Method		
		*E*_a_ (kJ/mol)	R^2^	*E*_k_ (kJ/mol)	R^2^	*A*_k_
A (+)	0.0256	79.8	0.9981	74.7	0.9978	3.68 × 10^3^
B (0)	0.0035	90.4	0.9763	84.7	0.9752	3.53 × 10^4^
C (−)	−0.0180	92.0	0.9983	86.2	0.9986	5.04 × 10^4^

## Data Availability

The datasets used and/or analysed during the current study are available from the corresponding author on reasonable request.
